# Optimal first‐line treatment for advanced thymic carcinoma

**DOI:** 10.1111/1759-7714.13181

**Published:** 2019-10-01

**Authors:** Xue Yang, Minglei Zhuo, Anhui Shi, Shengnan Yang, Ziping Wang, Meina Wu, Tongtong An, Yuyan Wang, Jianjie Li, Jia Zhong, Hanxiao Chen, Bo Jia, Zhi Dong, Jun Zhao

**Affiliations:** ^1^ Key Laboratory of Carcinogenesis and Translational Research (Ministry of Education), Department of Thoracic Medical Oncology Peking University Cancer Hospital and Institute Beijing China; ^2^ Key Laboratory of Carcinogenesis and Translational Research (Ministry of Education), Department of Radiation Oncology Peking University Cancer Hospital and Institute Beijing China; ^3^ Department of Geriatric Medicine The First Affiliated Hospital of Zhengzhou University Zhengzhou China

**Keywords:** Chemotherapy, prognosis, radiation therapy, resection, thymic carcinoma

## Abstract

**Background:**

Thymic carcinomas (TCs) are rare aggressive tumors with no standard first‐line treatment. This study was conducted to determine the optimal chemotherapy regimen for advanced TC.

**Methods:**

This retrospective study included 67 patients treated for stage IV TC in 2006–2015. The primary endpoints were the objective response rate (ORR) and progression‐free survival (PFS) with different chemotherapy regimens. Multivariate Cox regression analysis was used to identify factors associated with PFS, including metastatic status, radiotherapy post‐chemotherapy, primary lesion resection before chemotherapy, and chemotherapy regimen.

**Results:**

A total of 36 patients received a paclitaxel‐platinum regimen, 31 received a gemcitabine‐platinum regimen, 14 underwent primary lesion resection, and 33 underwent radiotherapy. ORR was 31% (11/36) and 29% (9/31) in the paclitaxel‐platinum and gemcitabine‐platinum groups, respectively (*P* = 0.890). Median PFS, one‐year PFS rate, and two‐year PFS rate were 7.0 months, 26%, and 6% with paclitaxel‐platinum treatment and 12 months, 48%, and 24% with gemcitabine‐platinum treatment (log‐rank *P* = 0.030). Median PFS, one‐year PFS rate, and two‐year PFS rate were 18.0 months, 57%, and 33% with surgical resection and 7.3 months, 31%, and 7% without resection (log‐rank *P* = 0.030). Median PFS, one‐year PFS rate, and two‐year PFS rate were 13.0 months, 52%, and 20% with radiotherapy and 4.3 months, 22%, and 7% without radiotherapy (log‐rank *P* = 0.001). In multivariate analysis, metastatic status (hazard ratio [HR], 0.33, *P* = 0.004), surgical resection (HR, 0.32; *P* = 0.004), and radiotherapy (HR, 0.32; *P* < 0.001) were associated with superior PFS.

**Conclusions:**

Both gemcitabine‐platinum and paclitaxel‐platinum regimens were efficacious for advanced TC. Primary lesion resection and radiotherapy may also benefit selected patients.

## Introduction

Although thymic tumors are the most common primary tumors in the anterior mediastinum, overall they are rare. They occur at a rate of only 1.5 cases for every million people each year in the US.^1^, ^2^ Thymic carcinoma is very rare, accounting for less than 1% of all tumors in the thymus. The data of incidence and prevalence of TC was deficient in China. The five‐year survival rate of thymic carcinomas (TCs) was approximately 40%.^3^, ^4^ TCs are derived from the thymic epithelium and include several histopathologic subtypes, such as squamous cell carcinoma, basaloid carcinoma, mucoepidermoid carcinoma, lymphoepithelioma‐like carcinoma, clear cell carcinoma, sarcomatoid carcinoma, adenocarcinomas, and undifferentiated carcinoma.^5^ Although a number of staging systems exist, the Masaoka‐Koga system remains the most commonly used system for TC.^1^, ^5^ Currently, chemotherapy with or without radiotherapy is recommended for advanced TC.^6^ Well‐established prognostic factors for TC include the extent of resection,^7^ as well as the stage and histologic type of tumor.^8^ Whether radiotherapy or chemotherapy influences prognosis remains controversial.^6^, ^7^, ^9^ The low incidence of TC hinders the ability to conduct large randomized clinical trials. Consequently, systemic treatment decisions are often guided by limited data from prospective trials, retrospective studies, and case reports.^10^ There is no standard first‐line chemotherapy regimen for advanced TC.

## Methods

The purpose of this study was to evaluate the optimal first‐line chemotherapy regimen in patients with advanced TC.

### Patient eligibility

A total of 67 patients with stage IV (including IVa and IVb) TC admitted to Beijing Cancer Hospital between January 2006 and December 2015 were recruited. The study was approved by the Hospital's Institutional Review Board. All patients provided written informed consent before collection and use of their clinical information.

The enrollment criteria were as follows: (i) diagnosis of TC based on the 2004 World Health Organization criteria, which was established by experienced pathologists; (ii) clinical stages IVa or IVb, according to Masaoka‐Koga staging system^11^; and (iii) complete follow‐up data until the study end date.

### Response evaluation and statistical analysis

The Response Evaluation Criteria in Solid Tumors (RECIST 1.1) system was used to evaluate tumor response. Objective response rate (ORR) was defined as the sum of the complete response (CR) and partial response (PR). Progression‐free survival (PFS) was defined as the length of time from the first cycle of chemotherapy to the time of documented tumor progression or death.

The primary endpoints of the study were the ORR and PFS of different chemotherapy regimens. Baseline clinical characteristics and ORRs of the two regimens were compared using Pearson's chi‐square test or Fisher's exact test. The following factors were analyzed to determine their effects on PFS: age, sex, metastatic status, radiotherapy after chemotherapy, resection of primary lesion before chemotherapy, type of chemotherapy regimen, and number of cycles of chemotherapy. The Kaplan‐Meier method was used to estimate median PFS, PFS rates at one year, and two years. Univariate analysis was conducted using the log‐rank test to identify prognostic factors for PFS. Significant factors (*P* < 0.05) in the univariate analysis were included in the multivariate Cox proportional hazards model. All *P‐*values were two‐sided, and *P* < 0.05 was considered significant. All statistical analyses were performed using SAS (SAS Institute Inc., Cary, NC, USA).

## Results

### Patient characteristics

A total of 67 patients with Masaoka‐Koga stage IV TC were enrolled in this study. Their clinical characteristics are summarized in Table 1. There were 42 men and 25 women, with a mean age at diagnosis of 53 years (range, 14–75 years). At the time of diagnosis, seven patients (10.4%) had stage IVa TC (only pleural or pericardial dissemination) and 60 patients (89.6%) had stage IVb TC. Among those patients with stage IVb disease, 18 had only lymph node metastasis, whereas 42 had distant metastasis involving one organ (*n* = 32) or more than one organ (*n* = 10). The extra‐lymphatic sites of metastasis included bone (19 patients), lungs (16 patients), liver (14 patients), and adrenal glands (three patients). Twenty‐seven patients had both lymph node and distant metastases. All patients received four to six cycles of chemotherapy.

**Table 1:** Baseline patient, tumor and treatment characteristics

Factor	Total (*n* = 67)	GEM (*n* = 31)	PTX (*n* = 36)	*P*‐value
Age, mean ± SD	53.0 ± 12.6	51.9 ± 14.3	53.9 ± 10.9	0.50†
Sex, *n* (%)				0.77‡
Male	42 (62.7)	20 (64.5)	22 (61.1)	
Female	25 (37.3)	11 (35.5)	14 (38.9)	
Age, *n* (%)				0.53‡
<65	52 (77.6)	23 (74.2)	29 (80.6)	
≥65	15 (22.4)	8(25.8)	7 (19.4)	
Pathological type				0.001‡
squamous carcinoma	64	31	33	
Adenocarcinoma	2	0	2	
Neuroendocrine carcinoma	1	0	1	
Metastasis, *n* (%)				0.022‡
Lymph node	18 (26.9)	12 (38.7)	6 (16.7)	
Distant	42 (62.7)	14 (45.2)	28 (77.8)	
None	7 (10.4)	5 (16.1)	2 (5.6)	
Masaoka‐Koga stage, *n* (%)				0.24§
IVa	7 (10.4)	5 (16.1)	2 (5.6)	
IVb	60 (89.6)	26 (83.9)	34 (94.4)	
Surgery, *n* (%)				0.36‡
No	53 (79.1)	23 (74.2)	30 (83.3)	
Yes	14 (20.9)	8 (25.8)	6 (16.7)	
Radiotherapy, *n* (%)				0.020‡
No	34 (50.7)	11 (35.5)	23 (63.9)	
Yes	33 (49.3)	20 (64.5)	13 (36.1)	

† Student's *t‐*test.

‡ Pearson's chi‐square test.

§ Fisher's exact test.

GEM, gemcitabine‐based chemotherapy; PTX, paclitaxel‐based chemotherapy; SD, standard deviation.

Patients were followed until 30 July 2018, with a median follow‐up period of 27.8 months (range, 4.5–88.7 months). By the time of last follow‐up, 58 patients had experienced progressive disease (PD) and 29 patients had died. The mortality rate was 43.2% (29/67).

### Chemotherapy

All patients received either a paclitaxel‐platinum regimen (*n* = 36; 53.7%) or a gemcitabine‐platinum regimen (*n* = 31; 46.3%) as their first‐line chemotherapy regimen. The paclitaxel‐based group patients received paclitaxel (175 mg/m^2^) plus cisplatin (75 mg/m^2^, day 1), carboplatin (area under the curve = 5, day 1), or nedaplatin (80 mg/m^2^, day 1) every three weeks for a maximum of six cycles. The gemcitabine‐based group patients were treated with gemcitabine (1250 mg/m^2^, days 1 and 8) plus cisplatin (75 mg/m^2^, day 1), carboplatin (area under the curve = 5, day 1), or nedaplatin (80 mg/m^2^, day 1) every three weeks for a maximum of six cycles. The distribution, PR, PD/SD (stable disease), and ORR of each regimen are shown in Table S1. No patient experienced a CR. ORR was not significantly different between the paclitaxel‐platinum and gemcitabine‐platinum regimens (30.6% and 29.0%, respectively; *P* = 0.890).

**Figure 1:**
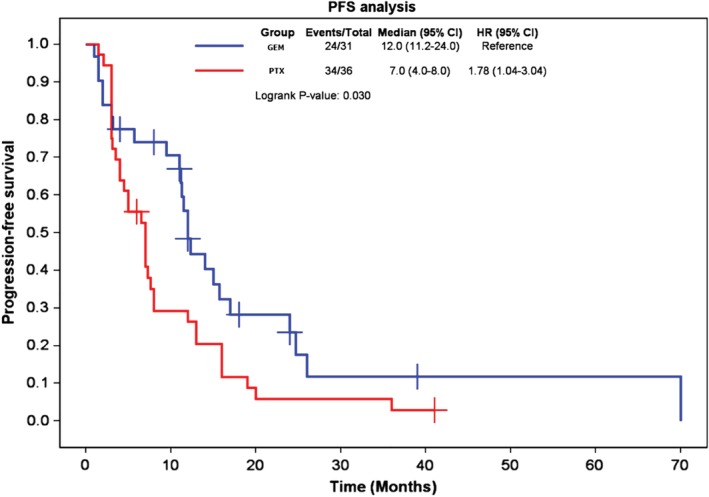
Kaplan‐Meier curves comparing progression‐free survival with the two chemotherapy regimens. GEM, gemcitabine‐based chemotherapy; PTX, paclitaxel‐based chemotherapy.

Figure 1 shows the effects of the two chemotherapy regimens on PFS. The median PFS, one‐year PFS rate, and two‐year PFS rate were 7.0 (95% confidence interval [CI], 4.0–8.0) months, 26%, and 6%, respectively, in the paclitaxel‐platinum group and 12 months (95% CI, 11.2–24.0), 48%, and 24% in the gemcitabine‐platinum group. PFS was superior with the gemcitabine‐platinum regimen (log‐rank *P* = 0.030).

### Surgical resection

**Figure 2:**
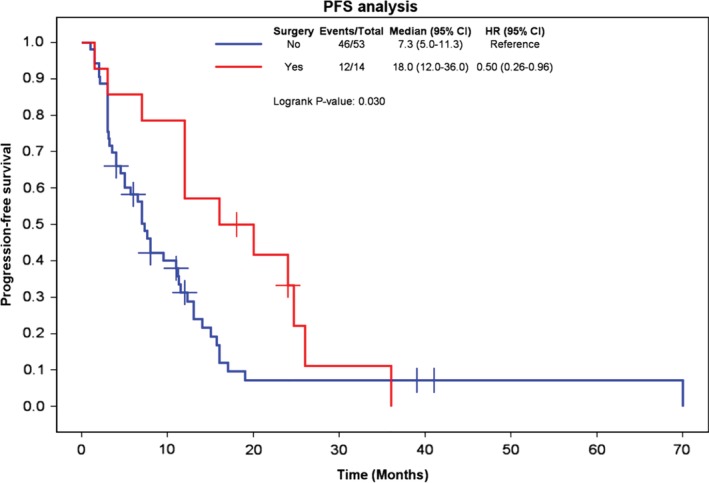
Kaplan‐Meier curves comparing progression‐free survival in patients who underwent surgical resection or not.

Only 14 patients (20.9%) underwent surgical resection before chemotherapy because resection was difficult to achieve. In four of these patients, the tumor was completely resected; in the other 10 patients, only palliative surgery was performed. Figure 2 shows the effects of surgical resection on PFS. The median PFS, one‐year PFS rate, and two‐year PFS rate were 18.0 months (95% CI, 12.0–36.0), 57%, and 33%, respectively, for patients who underwent resection and 7.3 months (95% CI, 5.0–11.3), 31%, and 7% for those who did not. PFS was superior with surgery (log‐rank *P* = 0.030).

### Radiotherapy

**Figure 3:**
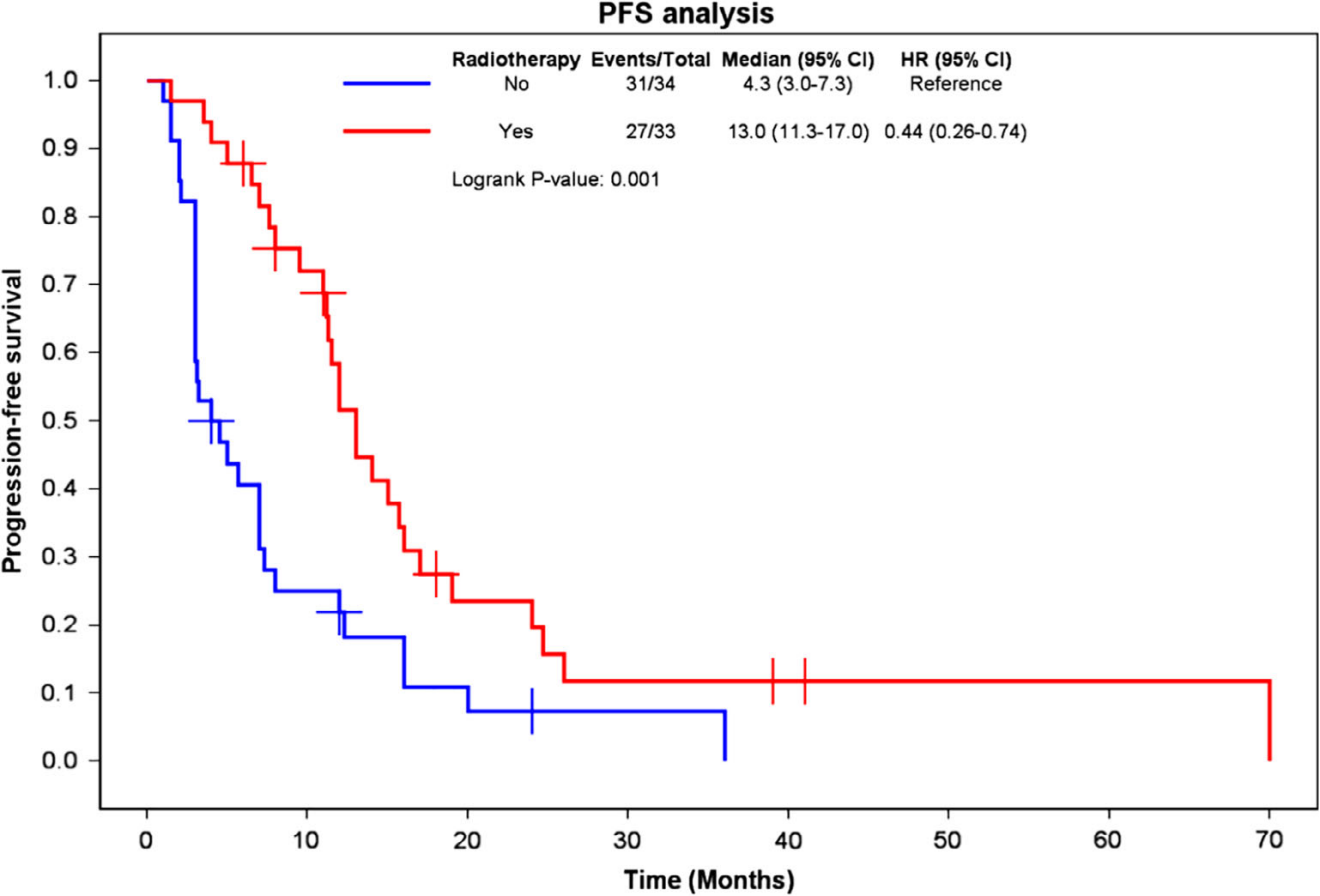
Kaplan‐Meier curves comparing progression‐free survival in patients who did or did not receive radiotherapy.

A total of 33 patients (49.3%) received radiotherapy after chemotherapy. Figure 3 shows the effect of radiotherapy on PFS. The median PFS, one‐year PFS rate, and two‐year PFS rate were 13.0 months (95% CI, 11.3–17.0), 52%, and 20%, respectively, in patients who received radiotherapy and 4.3 months (95% CI, 3.0–7.3), 22%, and 7% in those who did not. PFS was superior with radiotherapy (log‐rank *P* = 0.001).

### Tumor stage

Figure S1 shows the effect of tumor stage on PFS. The median PFS, one‐year PFS rate, and two‐year PFS rate were 26.0 months (95% CI, 12.3–36.0), 86%, and 51%, respectively, in patients with stage IVa TC and 7.6 months (95% CI, 6.5–12.0), 31%, and 9% in patients with stage IVb disease. PFS was not significantly different between these two groups (*P* = 0.080).

### Tumor metastasis

**Figure 4:**
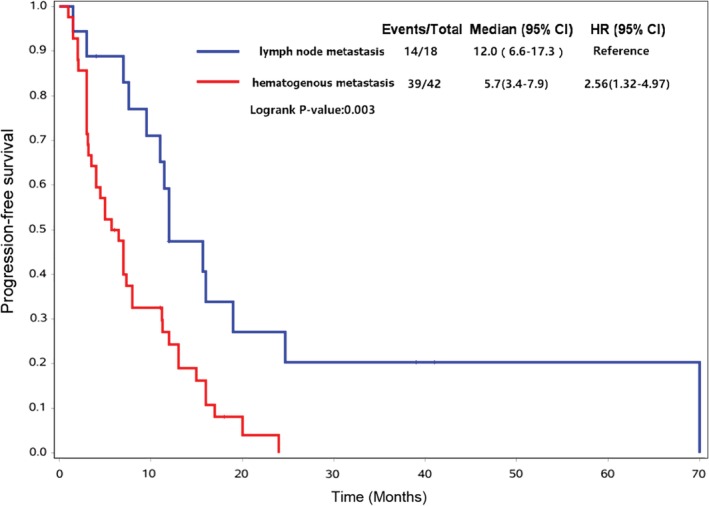
Kaplan‐Meier curves comparing progression‐free survival of patients with only lymph node metastasis and patients with hematogenous metastasis.

Figure 4 shows the PFS curves of patients with different metastasis status at the time of diagnosis. Median PFS was 12.0 months (95% CI, 6.6–17.3) in patients with only lymph node metastasis and 5.7 months (95% CI, 3.4–7.9) in those with distant metastasis. PFS was superior in patients with only lymph node metastasis (log‐rank *P* = 0.003).

### Multivariate analysis

In stepwise multivariate Cox regression analysis, surgical resection (hazard ratio [HR], 0.32; *P* = 0.005), radiotherapy after chemotherapy (HR, 0.32, *P* < 0.001), and metastatic status (HR, 2.63; *P* = 0.004) were independent prognostic factors for PFS (Table 2). The type of chemotherapy regimen was not significantly associated with PFS (*P* = 0.225). Age, sex, and number of cycles of chemotherapy were also not significantly associated with PFS.

**Table 2:** Evaluating influencing factors on progression‐free survial (PFS) by stepwise multivariate Cox regression mode

Variable	Regression coefficient	Standard error	*P*‐value	Hazard ratio	Lower HR	Upper HR
Surgery (yes vs. no)	−1.1228	0.3942	0.0049	0.32	0.15	0.70
Radiotherapy (yes vs. no)	−1.2327	0.3156	0.0001	0.32	0.16	0.54
Metastasis (distant vs. lymphatic only)	0.9684	0.3557	0.0044	2.63	1.31	5.29
Chemotherapy regimen (PTX vs. GEM)	−1.4336	1.1812	0.2249	0.24	0.02	2.41

## Discussion

In this study, which is one of the largest to evaluate chemotherapeutic efficacy and prognostic factors for advanced TC, we identified metastatic status, surgical resection before chemotherapy, and radiotherapy after chemotherapy as independent predictors of PFS.

Whether Masaoka‐Koga stage is an independent prognostic factor for advanced TC remains unclear. Engels and Pfeiffer^4^ Kelly^12^ and Masaoka *et al*.,^13^ considered Masaoka‐Koga stage to be a prognostic factor, whereas Cardillo *et al*.^14^ and Hosaka *et al*.^15^ did not. Although stage was not identified as an independent factor for PFS in our multivariate regression analysis, the PFS of patients with stage IVa TC was 26.0 months, which was much longer than the PFS of patients with stage IVb disease (7.6 months). We also found that distant metastasis was an independent prognostic factor for poor PFS; patients with lymph node metastasis alone had a superior PFS.

Many studies have confirmed surgical resection as the most important factor in the treatment of TC. Kondo and Monden reported five‐year survival rates of 67%, 30%, and 24% for total resection, subtotal resection, and inoperable treatment, respectively.^3^ Indeed, Yano *et al*. reported that resectability was the only prognostic factor for TC.^16^ In this study, we also demonstrated that PFS was superior in patients who underwent surgical resection (either total or palliative), compared with those who had inoperable tumors. Because of the highly aggressive behavior and frequent distant metastases seen with TC, it is difficult to achieve complete resection, especially for advanced tumors. Detterbeck *et al*. reported a low rate (25%) of R0 resection for stage IV thymomas and TCs.^8^ In the current study, only 14 (20.9%) of our patients underwent surgical resection, including total and palliative resections.

It has been previously reported that patients with advanced TC may benefit from radiotherapy.^17^, ^18^ Radiotherapy has long been established as an important component of therapy for this disease. Our results confirm its beneficial effects, as PFS was superior in patients who received radiotherapy after chemotherapy, compared with those who received no radiotherapy.

There is currently no standard first‐line chemotherapy regimen for advanced TC. Cisplatin‐ and adriamycin‐based regimens have been recommended by some authors, with response rates ranging from 42% to 50% and median PFS durations ranging from six to eight months.^19^, ^20^ In the latest version of National Comprehensive Cancer Network guidelines, a regimen of paclitaxel and carboplatin was recommended as first‐line chemotherapy for TC. This recommendation was based on a phase II study that reported an ORR of 21.7% and a median PFS of 5.0 months with paclitaxel‐carboplatin in patients with advanced TC.^21^ Retrospective studies reported ORRs of 22% to 38% and median PFS durations of five to nine months with this regimen.^22^, ^23^, ^24^ Our findings of an ORR of 30.6% and median PFS of seven months with the paclitaxel‐platinum regimen are consistent with prior results.

The literature is unclear regarding the efficacy of gemcitabine‐platinum regimens in TC. Only one study demonstrated that the regimen was effective in advanced TC, producing an ORR of 61.5% and a median PFS of 14.5 months.^25^ In the current study, the ORR of the gemcitabine‐platinum regimen was 29.03%, which was similar to that of the paclitaxel‐platinum regimen. In univariate analysis (log‐rank test), the median PFS of gemcitabine‐platinum regimen was 12 months, which was superior to that of the paclitaxel‐platinum regimen (seven months), but in multivariate analysis, the type of chemotherapy regimen was not associated with PFS. The discrepancy between univariate and multivariate analysis results likely reflects the differences in tumor stage and radiotherapy between the paclitaxel‐platinum and gemcitabine‐platinum groups. Despite the lack of clear superiority of the gemcitabine‐platinum regimen, our results indicate that it is an effective regimen for advanced TC, providing clinicians with another treatment option. Large‐scale prospective trials are warranted to determine the optimal chemotherapy regimen for TC.

The current study is limited by its retrospective nature and small sample size. However, because of the low incidence of TC, it is difficult to develop a large, prospective clinical study.

In conclusion, both gemcitabine‐platinum and paclitaxel‐platinum regimens demonstrated promising efficacy in advanced TC. Resection of the primary lesion before chemotherapy and radiotherapy after chemotherapy may be beneficial options for selected patients.

## Disclosure

No authors report any conflict of interest.

## Supporting information




**Figure S1.** Kaplan‐Meier curves comparing progression‐free survival of patients with stage IVa or stage IVb tumors.
**Table S1.** Distribution and response rates of the two chemotherapy regimens.
