# Supplementary material for: Optimal first‐line treatment for advanced thymic carcinoma
Source: Thorac Cancer. 2019 Oct 1;10(11):2081–7. doi: 10.1111/1759-7714.13181 (PMC6825903; doi:10.1111/1759-7714.13181)
Supplement: Supplementary file 1 — Figure S1. Kaplan‐Meier curves comparing progression‐free survival of patients with stage IVa or stage IVb tumors. Table S1. Distribution and response rates of the two chemotherapy regimens. [file TCA-10-2081-s001.doc]

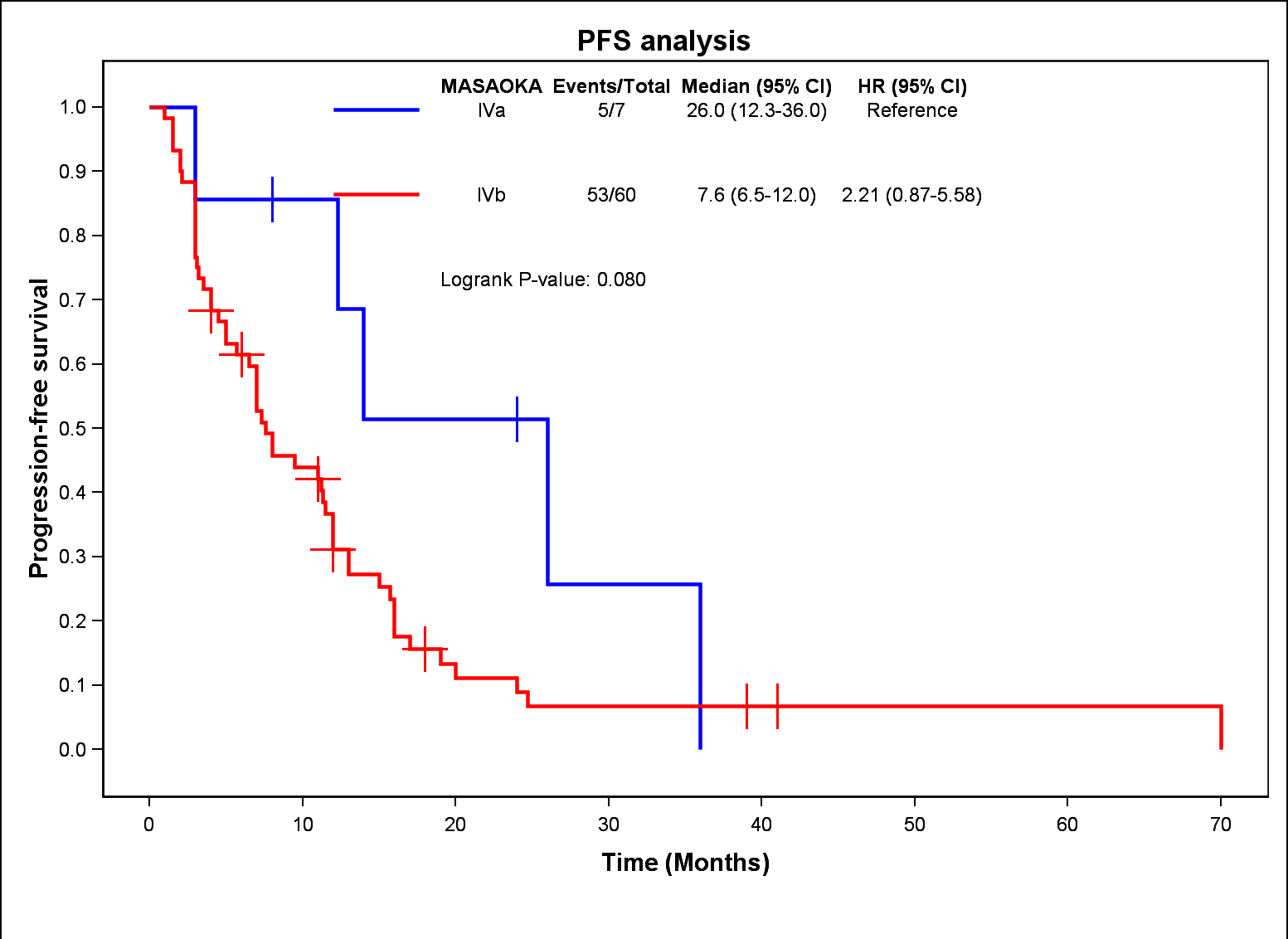
Supplement figure 1. Kaplan-Meier curves comparing progression-free survival of patients with stage IVa or stage IVb tumors.

**Supplement Table 1. Distribution and Response Rates of the Two Chemotherapy Regimens**

| **Regimen** | **Total**  **(N=67)** | **PR** | **PD/SD** | **ORR** | ***P* value** |
| --- | --- | --- | --- | --- | --- |
| **Paclitaxel–platinum** | 36 (53.7) | 11 | 25 | 30.6% | 0.890^a^ |
| **TP** | 11 (16.4) | 2 | 9 |
| **TC** | 23 (34.3) | 9 | 14 |
| **TN** | 2 (3.0) | 0 | 2 |
| **Gemcitabine–platinum** | 31 (46.3) | 9 | 22 | 29.0% |
| **GP** | 28 (41.8) | 9 | 19 |
| **GC** | 2 (3.0) | 0 | 2 |
| **GN** | 1 (1.5) | 0 | 1 |

^a^ Pearson's chi-square test

GC, gemcitabine plus carboplatin; GN, gemcitabine plus nedaplatin; GP, gemcitabine plus platinum; ORR, objective response rate (complete + partial response rate); PD, progressive disease; PR, partial response; SD, stable disease; TC, paclitaxel plus carboplatin; TN, paclitaxel plus nedaplatin; TP, paclitaxel plus platinum.
